# Neurological Manifestations in Hospitalized Geriatric Patients With COVID-19 at King Abdulaziz Medical City in Jeddah, Western Region, Saudi Arabia From 2020 to 2021: A Cross-Sectional Study

**DOI:** 10.7759/cureus.45759

**Published:** 2023-09-22

**Authors:** Yasir O Marghalani, Abdulrahman H Kaneetah, Muhammad A Khan, Ammar A Albakistani, Sultan G Alzahrani, Abdulbari Kidwai, Khalid W Alansari, Hamid S Alhamid, Muath H Alharbi, Ahmed Attar

**Affiliations:** 1 College of Medicine, King Abdullah International Medical Research Center, Jeddah, SAU; 2 College of Medicine, King Saud bin Abdulaziz University for Health Sciences, Jeddah, SAU; 3 Unit of Postgraduate Education, King Abdulaziz Medical City, National Guard Health Affairs, Jeddah, SAU; 4 Medical Education, King Saud bin Abdulaziz University for Health Sciences, Jeddah, SAU; 5 Department of Neuroscience, Ministry of National Guard Health Affairs, Jeddah, SAU; 6 Department of Medicine, Ministry of National Guard Health Affairs, Jeddah, SAU; 7 Department of Medicine, Hamilton Health Sciences, Hamilton, CAN; 8 Department of Medicine, McMaster University, Hamilton, CAN

**Keywords:** covid-19 neurological outcomes, sars-cov-2, covid-19 in geriatric patient, post-covid anosmia, stroke, intracerebral hemorrhage, covid delirium, length of hospital stay (los), covid-19

## Abstract

Introduction

COVID-19 involvement in the nervous system has been reported in many cases. Viral neuroinvasion has multiple routes of entry. Neurological manifestations of COVID-19 can be divided into ones of the central nervous system (CNS), such as headache, dizziness, altered mental status, ataxia, and seizure, and of the peripheral nervous system (PNS), including ageusia, anosmia, acute illness demyelinating polyneuropathy, and neuralgia.

Aim and objectives

This study aims to observe and report the neurological manifestations in geriatric patients who were diagnosed with COVID-19 at KAMC-J and report the duration of admission to the in-patient and ICU wards.

Methods

This was a cross-sectional study conducted on admitted geriatric patients with PCR-confirmed COVID-19 from April 1, 2020 to June 30, 2021 at KAMC-J. Using Raosoft®, the sample size was estimated with a CI of 95% and a 36.4% prevalence of neurological symptoms in COVID-19 patients to be 289. Convenience sampling was used, and the data were collected from BESTCare EMRs. IBM SPSS Statistics for Windows, Version 20 (Released 2011) was used for descriptive and inferential statistical analysis.

Results

In this study, a total of 290 patients’ data were collected, 161 (55.5%) of which were males. In addition, the median age was 71 (Q1-Q3: 65-78) years; furthermore, the median body mass index (BMI) was 30(Q1-Q3: 25-34) kg/m^2^. In descending order, the most prevalent comorbidities were hypertension (HTN) (70.3%), diabetes mellitus (DM) (68.6%), cardiac disease (42.1%), chronic kidney disease (26.6%), neurological disease (23.6%), cancer malignancy (13.1%), and finally chronic respiratory disease (11.4%). Regarding typical COVID-19 manifestations, 181 patients claimed to have experienced cough (62.4%), dyspnea by 164 (56.7%), fever by 154 (53.5%), fatigue by 93 (32.3%), a reading of anoxia by 68 (23.4%), abdominal pain by 58 (20.0%), diarrhea by 56 (19.4%), and finally throat pain by 19 (6.6%). Manifestations and pathologies of the CNS included headache (25.4%), dizziness (21.5%), impaired consciousness (17.2%), delirium (6.6%), ischemic stroke (4.1%), focal cranial nerve dysfunction (2.8%), seizure (2.8%), intracerebral hemorrhage (ICH) (0.3%), and ataxia (0.3%). Moreover, pathologies of the PNS manifested as taste impairment in 46 patients (15.9%), smell impairment in 33 (11.4%), nerve pain in 7 (24%), visual impairment in 5 (1.7%), Bell’s palsy in 2 (0.7%), and Guillain-Barre syndrome in 1 (0.3%). Moreover, the majority of patients who developed an ischemic stroke or ICH, or required admission to the ICU had either DM or HTN. In addition, 17 (25.4%) of the 67 patients admitted to the ICU developed impaired consciousness. All-cause mortality in our study was 31 (10.71%) cases.

Conclusion

Neurological manifestations of COVID-19 are common and can result in serious complications if not detected and managed early, especially in the elderly. These complications are mostly seen in severely ill patients and may be the only symptoms in COVID-19 patients. In addition, patients' clinical conditions could deteriorate rapidly and result in significant morbidity and mortality. Therefore, a high index of suspicion is required among healthcare providers when dealing with such cases. Moreover, we recommend systematically collecting data on the short- and long-term neurological complications of COVID-19 globally and documenting the functional long-term outcomes after these complications.

## Introduction

Coronavirus disease 2019 (COVID-19) is an infectious disease that initially emerged in December 2019 in Wuhan, China then globally causing the pandemic [1]. The disease’s spectrum of symptoms includes cough, anorexia, fatigue, shortness of breath, expectoration, myalgias and arthralgias, nausea, vomiting, and diarrhea among others. Neurological manifestations of the disease have been an area of interest. Some of these symptoms are self-limited when the disease is mild, like headaches and anosmia, while other severe symptoms that manifest with greater infection include disorders of consciousness and acute cerebrovascular disease. Moreover, severe acute respiratory syndrome coronavirus-2 (SARS-CoV-2) is caused by COVID-19, which is a life-threatening disease that has evolved to be a concern for global health [2]. Regarding its origin, COVID-19 is an RNA virus arising from bats and transmitted to humans through an unknown intermediary animal [3].

The disease spreads by inhalation of infected droplets or by contact where touching a contaminated surface and then touching the face may cause infection [4]. Furthermore, the general pathomechanism of neurological manifestations involves binding of viral surface S protein to Angiotensin-converting enzyme 2 (ACE2) receptors found on the blood-brain barrier (BBB) damaging it and subsequently allowing nervous tissue invasion. Another theory of entry involves retrograde axonal transport from the olfactory, respiratory, or enteric nerves that express. Once the virus reaches beyond the BBB, the virus binds to cells that express ACE2 receptors such as neurons, astrocytes, oligodendrocytes, and endothelial cells in the cerebral cortex, striatum, brain stem, choroid plexus, paraventricular nuclei of the thalamus, middle temporal gyrus and posterior cingulate gyrus [2]. Moreover, by involvement of the nervous system through cytokine storm syndrome, which is a state of continuous and uncontrolled inflammation marked by increased proinflammatory cytokines and increased leukocytes, induction is theorized to disrupt the BBB and subsequently expose the nervous system to reactive oxygen species and inflammatory markers [2]. Furthermore, the increased incidence of ischemic strokes and intracerebral hemorrhage (ICH) could be explained by the increased vascular permeability and increased complement and coagulation cascades mediated by increased interleukin-6 (IL-6) and D-dimer [2]. In addition, the disease incubation period is from 2 to 14 days where a patient may be asymptomatic and infectious at the same time. The majority of patients with COVID-19 have mild symptoms whereas patients who are elderly or have comorbidities such as heart failure or respiratory disorders might progress to acute respiratory distress syndrome (ARDS) or multi-organ failure [4,5].

COVID-19 involvement in the nervous system has been reported in many cases. Viral neuro-invasion has multiple routes of entry [2]. Neurological manifestations of COVID-19 can be divided into two categories. The first one is central nervous system (CNS) manifestations, such as headache, dizziness, cerebrovascular disease, impaired consciousness, altered mental status, cranial nerve dysfunction, ataxia, and seizure. The second category is peripheral nervous system (PNS) manifestations, including ageusia, anosmia, visual impairment, and neuralgia. Others like musculoskeletal manifestations have also been reported, such as rhabdomyolysis. Rare occurrences have been documented of optic neuritis, encephalitis, and Guillain-Barré syndrome (GBS) [6].

One study that was published in 2020 in The Journal of the American Medical Association of Neurology included 214 patients with polymerase chain reaction (PCR) positive tests for SARS-CoV-2 [2]. Approximately one-third or 78 (36.4%) of patients had neurological manifestations of the disease. Of those with neurological manifestations, (67.9%) had central nervous system symptoms, most commonly dizziness (46.2%) and headache (35.8%). Moreover, (24.4%) had peripheral nervous system manifestations, including impaired taste (15.4%) and smell (14.1%). In addition, the same previous study also reports that (6.5%) had cerebrovascular diseases, (17.2%) had impaired consciousness, and (24.1%) had skeletal muscle injury [6]. A systematic review of 152 published studies in 2020 conducted in 23 countries on 41,409 individuals shows that only 20.82% had neurological manifestations [7]. Another study that was conducted on 841 patients hospitalized with COVID-19 developed neurological symptoms, of those manifestations, headache (14.1%), dysgeusia (6.2%), dizziness (6.1%), anosmia (4.9%), cerebrovascular diseases (1.7%), seizures (0.7%) [8]. All of these studies point out that neurological symptoms are common and may be serious.

To provide evidence to guide clinical decision-making in anticipating and promptly managing neurological complications, this study aims to closely observe the presentation and course of COVID-19 infection in hospitalized patients focusing on neurological symptoms at King Abdulaziz Medical City in the Western region of Jeddah from April 2020 to June 2021.

## Materials and methods

This research is a cross-sectional study that aims primarily to assess the neurological manifestations of hospitalized geriatric patients with COVID-19 or SARS-CoV-2, at King Abdulaziz Medical City, Jeddah, Saudi Arabia from April 2020 to June 2021. In addition, secondary objectives are to measure the total length of stay in both wards and ICU, report the number of ICU admissions, and assess comorbidities that contributed to necessitate an ICU admission. Using Raosoft® at http://www.raosoft.com/samplesize.html, the sample size was estimated with a CI of 95% and a prevalence of 36.4% [6] neurological symptoms in COVID-19 patients to be 289 (Raosoft). The convenience sampling technique was used, and the inclusion criteria were being hospitalized above 60 years old diagnosed with COVID-19 by PCR and experiencing neurological manifestations between April 1, 2020 and June 30, 2021 at KAMC-J. In addition, exclusion criteria were being younger than 60 years old and having a confirmed causative pathogen other than COVID-19. Furthermore, the patients’ data were collected from BESTCare electronic medical records (EMRs) using a data collection sheet which included the neurological manifestations of COVID-19 patients such as headache, dizziness, cerebrovascular disease, transient impaired level of consciousness that does not meet the criteria for delirium, seizures, ageusia, anosmia, and visual impairment. Furthermore, typical symptoms of COVID-19 were collected such as sore throat, fever, and cough. Demographic variables such as age, gender, and weight were collected as well. Moreover, the collection sheet contained a section for patient comorbidities such as diabetes, hypertension (HTN), and neurological diseases such as neurocognitive disorders, multiple sclerosis, amyotrophic lateral sclerosis, past cerebral vascular accident, and epilepsy.

Descriptive analysis was used, by using IBM SPSS Statistics for Windows, Version 20 (Released 2011; IBM Corp., Armonk, New York, United States). Categorical data were presented as frequency and percentage, and continuous data were depicted in the form of mean and standard deviation. The chi-square test was used to compare categorical variables like the neurological manifestations with demographics in patients with COVID-19. A p-value lower than 0.05 was considered significant.

Ethical consideration 

Data collection for this study was approved by the Institutional Review Board (IRB) at the King Abdullah International Medical Research Center, Jeddah, Saudi Arabia. The IRB waived the need for the patient’s consent because of the retrospective design.

## Results

In this study, a total of 290 patients’ data were collected, 161 (55.5%) of which were males. In addition, the median age was 71 (Q1-Q3: 65-78) years; furthermore, the median body mass index (BMI) was 30 (Q1-Q3: 25-34) kg/m^2^. A summary of patients’ demographics and comorbidities is presented in Table 1 with age and BMI being presented in Table 2. In descending order, the most prevalent comorbidities were HTN in 204 patients (70.3%), diabetes mellitus (DM) in 199 patients (68.6%), cardiac disease in 122 patients (42.1%), chronic kidney disease (CKD) in 77 patients (26.6%), neurological diseases in 68 patients (23.6%), cancer malignancy in 38 patients (13.1%), and finally chronic respiratory disease in 33 patients (11.4%). All-cause mortality in our study was 31 (10.71%) cases.

**Table 1:** Baseline Demographic Characteristics CKD: Chronic kidney disease; DM: diabetes mellitus

Medical History		n=290	%
Gender	Female	129	44.5
	Male	161	55.5
CKD (n=289)	No	212	73.4
	Yes	77	26.6
DM	No	91	31.4
	Yes	199	68.6
Hypertension	No	86	29.7
	Yes	204	70.3
Cardiac Diseases	No	168	57.9
	Yes	122	42.1
Neurological Diseases	No	220	76.4
	Yes	68	23.6
Chronic Respiratory Diseases	No	257	88.6
	Yes	33	11.4
Malignancy	No	252	86.9
	Yes	38	13.1
All Cause Mortality	No	259	89.31
	Yes	31	10.69

**Table 2:** Quantitative Demographics and Lengths of Stay (LoS) BMI: Body mass index; ICU: intensive care unit; LoS: length of stay

Variable	N	Median	IQR	Min	Max
Age	290	71	65 - 78	55	99
BMI	289	30	25 - 34	16	71
ICU LoS	66(23.1%)	6.5	2 - 17	1	114
In-patient LoS	286	9	4 - 14	1	114

Table 3 presents the COVID-19-related manifestations found in our patients. From most common to least, 181 patients claimed to have experienced cough (62.4%), 164 patients experienced dyspnea (56.7%), 154 patients experienced fever (53.5%), 93 patients experienced fatigue (32.3%), and 68 patients experienced anoxia 68 (23.4%), 58 patients experienced abdominal pain (20.0%), 56 patients experienced diarrhea (19.4%), and finally 19 patients experienced throat pain (6.6%).

**Table 3:** COVID-19 Typical Manifestations

Typical manifestations		n=290	%
Cough	No	109	37.6
	Yes	181	62.4
Anoxia	No	222	76.6
	Yes	68	23.4
Diarrhea (n=289)	No	233	80.6
	Yes	56	19.4
Throat Pain	No	271	93.4
	Yes	19	6.6
Abdominal Pain	No	232	80.0
	Yes	58	20.0
Fatigue (n=288)	No	195	67.7
	Yes	93	32.3
Dyspnea (n=289)	No	125	43.3
	Yes	164	56.7
Fever (n=289)	No	134	46.5
	Yes	154	53.5

**Figure 1:**
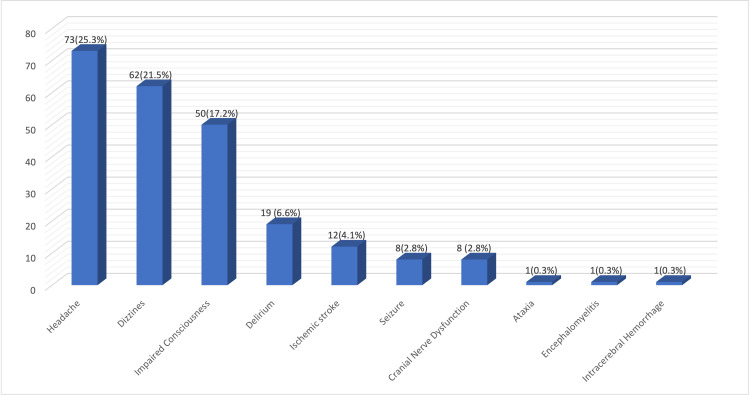
Central Nervous System Manifestations

Moreover, Figure 1 shows a summary of the manifestations and pathologies of the CNS which included headache in 73 patients (25.4%), dizziness in 62 (21.5%), impaired consciousness in 50 (17.2%), delirium in 19 (6.6%), ischemic stroke in 12 (4.1%), focal cranial nerve dysfunction in 8 (2.8%), seizures in 8 (2.8%), ICH in 1 (0.3%), ataxia in 1 (0.3%), and encephalopathy in 1 (0.3%).

**Figure 2:**
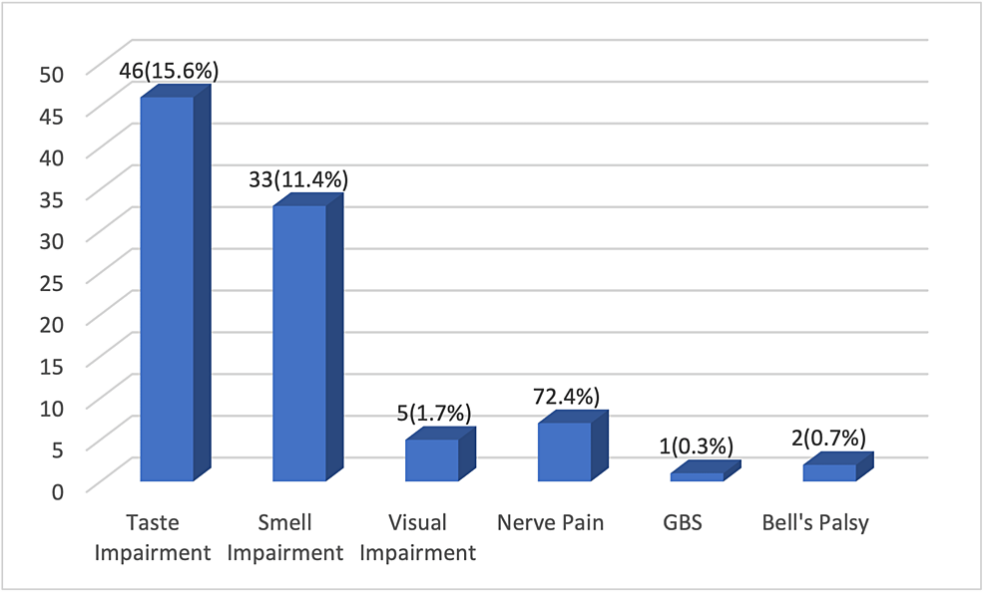
Peripheral Nervous System Symptoms GBS: Guillain-Barre Syndrome

Furthermore, Figure 2 illustrates symptoms and pathologies of the PNS. Taste impairment in 46 patients (15.9%), smell impairment in 33 (11.4%), nerve pain in 7 (24%), visual impairment in 5 (1.7%), Bell’s palsy in 2 (0.7%), and GBS in 1 (0.3%) were reported.

In relation to DM, it was present in 8 (66.7%) of 12 who had developed ischemic stroke, in one patient (100%) who suffered from ICH, and in 45 (67.2%) of 66 who were admitted to the ICU. In addition, HTN was present in 9 (75%) of 12 patients who developed an ischemic stroke, in 1 (100%) had ICH, and in 51 (76.1%) of 66 patients who required ICU admission. The aforementioned associations are presented in Table 4.

**Table 4:** Associations of DM and HTN With Ischemic Stroke, ICH, and ICU Admission DM: Diabetes mellitus; ICU: intensive care unit

Complication		DM				p-value
		No		Yes		
		n=91	%	n=199	%	
Ischemic stroke	No	87	31.30%	191	68.70%	>0.99
	Yes	4	33.30%	8	66.70%	
Intracerebral hemorrhage	No	91	31.50%	198	68.50%	0.686
	Yes	0	0.00%	1	100.00%	
ICU was required	No	69	30.90%	154	69.10%	0.770
	Yes	22	32.80%	45	67.20%	
Complication		HTN				p-value
		No		Yes		
		n=86	%	n=204	%	
Ischemic stroke	No	83	29.90%	195	70.10%	0.72
	Yes	3	25.00%	9	75.00%	
Intracerebral hemorrhage	No	86	29.80%	203	70.20%	0.7
	Yes	0	0.00%	1	100.00%	
ICU was required	No	70	31.40%	153	68.60%	0.24
	Yes	16	23.90%	51	76.10%	

As illustrated in Table 5, regarding impaired consciousness, 17 (25.4%) of the 67 patients admitted to the ICU suffered from impaired consciousness (OR 1.958; 95% CI 1-3.8, p-value 0.044). Moreover, 5 (41.7%) of the patients who suffered from an ischemic stroke 12 have been reported to have impaired consciousness (OR 3.698; 95% CI 1.12-12.17; p-value 0.038). Furthermore, with regard to seizures as presented in Table 6, five (62.5%) of the eight patients who had a seizure had a history of a neurological disease (OR 5.71; 95% CI; p-value 0.02). 

**Table 5:** Associations of Impaired Consciousness With ICU Admission and Ischemic Strokes CI: Confidence interval; ICU: intensive care unit

Complication		Impaired Consciousness				OR	95% CI	p-value
		No		Yes				
		n=240	%	n=50	%			
ICU Admission	No	190	85.2	33	14.8	1.958	1-3.8	0.044
	Yes	50	74.6	17	25.4			
Ischemic Stroke	No	233	83.8	45	16.2	3.698	1.12-12.17	0.038
	Yes	7	58.3	5	41.7			

**Table 6:** Association of Neurological Disease Presence With Seizures CI: Confidence interval

Complication		Neurological Disease				OD	95% CI	p-value
		No		Yes				
		n=219	%	n=68	%			
Seizure	No	216	77.4	63	22.6	5.714	1.33-24.57	0.02
	Yes	3	37.5	5	62.5			

## Discussion

The significant risk of developing neurological symptoms in COVID-19 patients has been confirmed by earlier research on COVID-19 in addition to other coronaviruses, such as the Middle East respiratory syndrome coronavirus (MERS-CoV) and SARS-CoV [2]. As explained previously, COVID-19 has multiple mechanisms of affecting the nervous system through direct entry and inducing an inflammatory state [2]. Moreover, COVID-19 patients have a generally high prevalence of neurological complications, which may lengthen their hospital stays and delay their recovery. Furthermore, some manifestations may result in significant morbidity and mortality more so in the geriatric population. Some manifestations need to be anticipated to ensure appropriate prevention and prompt intervention. 

Regarding patients’ comorbidities, the most prevalent diseases in our sample were hypertension in 204 patients (70.3%), DM in 199 (68.6%), and cardiac disease in 122 (42.1%), which is similar to what Singhal et al. found where hypertension (48, 95% CI- 36-60% I2-92%), diabetes mellitus (22, 95% CI- 13-32%, I2-86%), and cardiovascular disease (19, 95% CI - 11-28%, I2-85%) were the most reported comorbidities [9]. Moreover, regarding COVID-19-related symptoms, the commonest symptoms were cough in 181 patients (62.4%), dyspnea in 164 (56.7%), and fever in 154 (53.5%) which is similar to Singhal et al. systematic review findings where the most common symptoms were fever (83, 95% CI- 66-97%, I2-91%), cough (60, 95% CI- 50-70%, I2-71%), and dyspnea (42, 95% CI- 19-67%, I2-94%) [9].

In line with what Niazkar et al. concluded, headache was found in 73 (25.3%) of our participants and in 6.5% to 23% of the review article by Niazkar et al. and the mean prevalence of 8% in different studies [10]. Moreover, 62 participants commonly reported dizziness (21.5%), which is similar to what Mao et al. case series found dizziness in 36 (16.8%) admitted patients [6]. In addition, symptoms of significance in admitted elderly are impaired consciousness, or confusion, and delirium which were present in 50 (17.2%) and 19 (6.6%) of our patients, respectively. The number of patients who experienced confusion exceeded the 9% of the figure reported by Chen et al. which may be explained by the fact that their study observed all age groups while our study focused on the geriatric age group which is susceptible to such manifestations due to presence of comorbidities and subsequently having increased severity of disease [11,12]. Furthermore, Poloni et al. reported Delirium-Onset COVID-19 (DOC) in 21 (36.8%) elderly patients which is greater than our finding. To elaborate, this difference may be explained by the fact that participants of the study mentioned had an older mean age of 82.2 years, with more than three concomitant diseases in 18 (31.6%), and all lived in a dementia facility [13].

With regard to pathologies of the peripheral nervous system, the most common were ageusia or hypogeusia 46 (15.9%), anosmia, or hyposmia 33 (11.4%), and nerve pain 7 (2.4%). Regarding taste and smell impairment, Sheffah et al. found that smell impairment and taste impairment were present in 162 (58.47%) and 148 (53.4%) of the 277 subjects, respectively [14]. The possible explanations for this disparity are underreporting by older patients and that their sample mean age was younger 42.81 ± 16.76 years; moreover, according to Sehanobish et al. older patients are less likely to notice anosmia and ageusia due to age-related decrease in baseline olfactory and gustatory functions [15].

According to Al Kuwari et al., patients with either DM or HTN had a more severe clinical course [16]. In this study, 8 (66.7%) and 9 (75%) of 12 patients who developed an ischemic stroke had concomitant DM and HTN, respectively. To elaborate, in addition to baseline endothelial dysfunction associated with HTN [17] and diabetes [18], COVID-19 induces significant cytokine release, infection-associated endothelial dysfunction, profound inflammation, and an overall hypercoagulable state. These factors may have predisposed these patients to develop ischemic strokes [19]. Another severe manifestation was an ICH that presented in a patient with both DM and HTN [20]. Similarly, Dogra et al. reported that HTN and DM were present in 16 (48.5) and 10 (30.3) of ICH cases, respectively. To clarify, they hypothesize that the majority of ICHs were a result of an existing ischemic stroke, which is associated with both DM and HTN [20].

Regarding the hospital length of stay (LoS), this study’s results, median stay inwards for 9 days (Q1-Q3:4-14), are equal to what was reported by Alimohammadi et al. where the pooled median of LoS is 9 days [21]. However, a case of GBS with COVID-19 (GBS-C+) was an outlier with a LoS of 112 days which may be due to developing respiratory failure, requiring ICU admission, and invasive mechanical ventilation which is in harmony with what Ahmad et al. concluded [22]. Moreover, the majority of patients admitted to the ICU had DM or HTN or both; however, it was statistically insignificant. This agrees with what Al Kuwari et al. concluded stating that DM and HTN are associated with increased disease severity with ICU admission being an identifier and subsequently requiring longer LoS [16]. Knowing the LoS or other adverse events in advance can help the healthcare systems organize the allocation of limited resources more efficiently.

Limitations

This study's cross-sectional design makes it susceptible to the design's inherent limitations; one example is that the time period this study was done may not reflect the nature of the ever-mutating disease in the future. Moreover, cross-sectional studies are not the best in demonstrating causality. Furthermore, this study utilized convenience sampling which imposes a risk of selection bias. Last but not least, since this study focused on the geriatric population within one hospital, it might not accurately reflect the prevalence of neurological complications in COVID-19 patients in the general geriatric population. 

## Conclusions

Neurological manifestations of COVID-19 are common and can result in serious complications if not detected and managed early. In addition, patients' clinical conditions could deteriorate rapidly and result in significant morbidity and mortality. Therefore, a high index of suspicion is required among healthcare providers when dealing with such cases for prompt treatment and prevention of mentioned clinical manifestations. Moreover, we recommend systematically collecting data on the short- and long-term neurological complications of COVID-19 from different parts of the world and documenting the functional long-term outcomes after these complications.
